# Anti-inflammatory, antioxidant and anti-virulence roles of atractylodin in attenuating *Listeria monocytogenes* infection

**DOI:** 10.3389/fimmu.2022.977051

**Published:** 2022-10-28

**Authors:** Lei Xu, Yonglin Zhou, Jingwen Xu, Xiangzhu Xu, Gejin Lu, Qianghua Lv, Lijuan Wei, Xuming Deng, Xue Shen, Haihua Feng, Jianfeng Wang

**Affiliations:** ^1^ Department of Respiratory Medicine, Center for Pathogen Biology and Infectious Diseases, Key Laboratory of Organ Regeneration and Transplantation of the Ministry of Education, The First Hospital of Jilin University, Changchun, China; ^2^ State Key Laboratory for Zoonotic Diseases, Key Laboratory for Zoonosis Research, Ministry of Education, College of Veterinary Medicine, Jilin University, Changchun, China; ^3^ Changchun Veterinary Research Institute, Chinese Academy of Agricultural Sciences, Changchun, China; ^4^ Hebei Veterinary Medicine Technology Innovation Center, Shijiazhuang, China; ^5^ Department of Food Science, College of Food Science and Engineering, Jilin University, Changchun, China

**Keywords:** atractylodin, *Listeria monocytogenes*, listeriolysin O, NLRP3, Nrf2

## Abstract

**Background:**

*Listeria monocytogenes* (*L. monocytogenes*), as a pandemic foodborne pathogen, severely threatens food security and public health care worldwide, which evolves multiple bacterial virulence factors (such as listeriolysin O, LLO) for manipulating the immune response of *L. monocytogenes*-host interactions.

**Methods:**

Hemolysis assay was employed to screen a potential LLO inhibitor and the underlying mechanisms were investigated using molecular dynamics (MD) simulation and oligomerization assay. The effects of candidates on immune response were examined by qRT-PCR and immunoblotting analysis. Histological analysis, ELISA assay and biochemistry detection were conducted to assess *in vivo* efficacy of candidates.

**Results:**

In the present study, natural terpenoid atractylodin was characterized as an alternative drug candidate for the treatment of *L. monocytogenes* by the regulation of LLO function and host Nrf2/NLRP3 signaling pathway. Notably, *in vivo* infection model by *L. monocytogenes* also highlighted that atractylodin treatment provided effective therapeutic benefits, as evidenced by decreased bacterial burden and diminished inflammation. Congruently, the survival rate of *L. monocytogenes*-infection mice increased significantly from 10.0% to 40.0% by atractylodin treatment.

**Conclusion:**

Collectively, our study showed for the first time that atractylodin has tremendous potential to attenuate *L. monocytogenes* pathogenicity by blocking LLO pore formation and mediating the suppression of inflammation and oxidative stress, providing a promising therapeutic strategy and broadening the applications of atractylodin against *L. monocytogenes* infection.

## Introduction

*Listeria monocytogenes* is recognized as a crucial zoonotic foodborne gram-positive opportunistic bacterium which is the pathogenic bacteria of listeriosis, manifested in bacteremia, meningitis, abortion, and even fetal infections in human or animals (1). *L. monocytogenes* is a highly versatile psychrophilic and heat-resistant microorganism and possesses the ability to survive and multiply in different substrates, which thrives in acidic environments and high concentrations of sodium chloride and other salts (2, 3). Hence, the regulation of *L. monocytogenes* virulence is one of the most pressing problems for food safety and public health security, and it underlines the need to improve the control of bacterial virulence.

With the emergence and spread of antibiotic-resistant bacteria, the clinical efficacy of antibiotics against clinical pathogenic bacterial infections were seriously impaired. Therefore, feasible therapeutic strategies are critically needed to attenuate *L. monocytogenes* virulence. As an intracellular bacterium and important model pathogen, *L. monocytogenes* secretes a variety of virulence factors, which mediate cytotoxicity and contribute to the cell invasion (4). Listeriolysin O (LLO), a member of the cholesterol-dependent cytolysin (CDC) family, has proven to be important for *L. monocytogenes* in the progress of the destabilization and disruption of phagocytic lysosomes and the reproduction in the cytoplasm (5). Moreover, the virulence of LLO-defective strains declined evidently compared to the wild-type strains (5). Consistent with the finding, targeting for LLO function could be served as an important and novel therapy strategy against *L. monocytogenes* infections.

Nuclear factor erythroid 2-related factor 2 (Nrf2), as a transcription factor, plays a crucial role in cellular survival and defense against various stresses through antioxidant gene regulation, including classical enzymes (superoxide dismutases (SODs), catalase, and glutathione peroxidase (GPx)), typical phase 2 detoxifying enzymes (glutamate-cysteine ligase catalytic subunit (GCLC), glutamate-cysteine ligase modifier subunit (GCLM) and NADP(H):quinone oxidoreductase-1 (NQO1)), the stress response protein heme oxygenase-1 (HO-1) (6). Recent evidences emphasized the beneficial role of Nrf2 in bacterial infection (7). Intriguingly, Nrf2 activation contributed to the suppression of NLRP3 (NOD-, LRR- and pyrin domain-containing protein 3) inflammasome activation in macrophages, suggesting that activation of Nrf2 is a potential therapeutic strategy for NLRP3-associated inflammatory diseases. NLRP3 inflammasomes consist of the NLRP3 scaffold, ASC adaptor, and pro-caspase-1 (8) activated by *L. monocytogenes* infection effectively rely on an LLO-mediated phagosomal disruption (9). The pivotal role of the NOD-like receptor (NLR) family in the immune system has gained attractive attention in recent years, among which the partakes in the progress of usual (auto) inflammatory diseases by activating caspase-1 and cracking the pro-inflammatory mediators, which induce inflammation upon secretion (10).

Notably, various compounds present in medicinal herbs, vegetables and fruits have been found to induce anti-inflammatory and antioxidant activities and used as dietary supplements or therapeutic candidates. Among the abundance of natural compounds, an active polyethylene alkyne extracted from the rhizome of *Atractylodes chinensis*, atractylodin exhibits a wide range of pharmacological activities including antioxidant, anti-inflammatory and hypoglycemic properties. Atractylodin could decrease the level of interleukin-6 (IL-6) by inhibiting MAPKs activation in HMC-1 cells (11, 12), and attenuate LPS-induced acute lung injury by inhibiting NLRP3 inflammasome and TLR4 signal pathways (13). To date, the potential effects of atractylodin against the *L. monocytogenes* infection has not yet been explored. In the current study, we probed into the key mechanisms underlying the therapeutical effect of atractylodin against *L. monocytogenes* infection *via* simultaneously modulating the bacterial virulence and regulating the immune response of *L. monocytogenes*-host interactions. Inspiringly, our findings demonstrated that atractylodin treatment could prominently attenuate *L. monocytogenes* virulence by the inhibition of LLO function and the regulation of Nrf2/NLRP3 signaling pathway, which provided a promising drug candidate and a feasible therapeutic strategy to combat *L. monocytogenes* infection.

## Materials and methods

### Bacterial strains and reagents

The wild-type *L. monocytogene* strain EGD, the LLO deletion mutant EGDΔ*hly* and its complementation strain EGDΔ*hly*::*hly* were used in this study. Bacteria were grown at 37°C in Trypticase Soy Broth (TSB, Qingdao Hope Biol-Technology Co.,Ltd) medium with or without the indicated concentrations of atractylodin dissolved in dimethylsulfoxide (DMSO, Sigma). The atractylodin (≥ 98%) used in the current study was purchased from Chengdu Herbpurify Biotechnology Co., Ltd.

The antibodies used in the present study were as follows: Nrf2 (Immunoway, TX, USA); Keap1, NQO1, NLRP3, Caspase-1, ASC (Cell Signal Technology, MA, USA); β-actin, HRP-conjugated goat anti-rabbit, goat anti-mouse, donkey anti-goat secondary antibodies (Proteintech, MA, USA); LLO (Abcam, MA, USA); HO-1 (Santa Cruz, CA, USA); IL-1β (R&D Systems, Minn, USA); Lamin B (Novus Biologicals, CO, USA); ICDH (14). Additionally, AST, ALT, MPO, SOD and Potassium Assay Kits were purchased from the Nanjing Jiancheng Bioengineering Institute (Nanjing, China). The enzyme-linked immunosorbent assay (ELISA) MAX™ Deluxe Set were obtained from BioLegend (CA, USA).

### Animals and cell culture

Female Balb/C mice and wild-type C57BL/6 mice (6-8 weeks, weighting approximately 18-20 g each) were obtained from Liaoning Changsheng Biotechnology Co., Ltd (Liaoning, China). C57BL/6 mice with Nrf2^−/−^ (Knockout) were obtained by the Jackson Laboratory (Bar Harbor, ME, USA). Animals were provided adequate food and water with the relative humidity of 55 ± 10% at room temperature. All the animal assays were approved by the Animal Welfare and Research Ethics Committee in Jilin University.

Mouse macrophagelike cell line J774A.1 were cultured in Dulbecco’s modified eagle medium (DMEM) containing 10% fetal bovine serum (Biological Industries, BI), penicillin (100 units/mL) and streptomycin (100 μg/mL) (Medical Research Council, MRC) at 37°C with 5% CO_2_ atmosphere.

For mouse peritoneal macrophage preparation, C57BL/6 mice were stimulated with Difco™ Fluid Thioglycollate Medium *via* intraperitoneal injection and kept in aseptic conditions for 4 days. Peritoneal macrophage were harvested by washing the peritoneal lavage of mice with RPMI-1640 medium and centrifuging at 1,000 r.p.m. for 10 min. And then the peritoneal macrophage were re-suspended in RPMI-1640 medium with 10% FBS. Mouse peritoneal macrophages were seeded into 6-well plates or 96-well plates and incubated at 37°C in 5% CO_2_ humidified air.

### Hemolysis inhibition assay

Bacterial culture supernatants or recombinant LLO was firstly incubated with different concentrations of atractylodin at 37°C for 20 min in PBS (35 mM Na_2_HPO_4_·12H_2_O, 125 mM NaCl, pH 5.5). Subsequently, rabbit red blood cells (RBCs) were added to each sample and co-incubated at 37°C for another 20 min. Then, the erythrocytes were separated by centrifugation. Samples treated with ddH_2_O were completely hemolytic considered as 100% lysis controls. Ultimately, the absorbance of supernatant was measured at OD_570nm_ by a microplate reader to calculate the percentage of hemolysis.

### Bacterial growth and LLO expression analysis

For the growth curve assay, atractylodin with the final concentrations of 0, 1, 2, 4 and 8 µg/mL was added to *L. monocytogenes* EGD culture medium. Then, the mixture samples were further cultured at 37°C with shaking at 200 rpm, and the absorbance was measured by reading at OD_600nm_ at 30-min intervals to analyze the effect on bacterial growth.

Until the OD_600nm_ remained stable, culture supernatants and precipitate were collected by centrifugation (12,000 g, 10 min) and boiled with 5× loading buffer at 100°C for 10 min for further western blotting analysis, and ICDH was used as an internal control.

Furthermore, *E. coli* BL21(DE3)-pET21a-LLO obtained in our lab was cultured to OD_600nm_ = 0.6-0.8, induced by isopropyl-β-D-thiogalactopyrandoside (IPTG, 1 mM) mixed with different concentrations of atractylodin (0, 4, 8 μg/mL) at 16°C overnight. Then, the isopyknic bacteria suspension was ultrasonic cracked, centrifuged and collected to boil with 5 × loading buffer at 100°C for SDS-PAGE analysis. And the protein of each sample was stained by coomassie brilliant blue.

### Oligomerization assay

LLO was preincubated with or without the indicated concentrations of atractylodin at 37°C for 20 min, and LLO oligomerization was induced *in vitro* as previously describe (15). Then, the oligomers and monomers were determined with an anti-His tag antibody (ABclonal, Wuhan, China) by western blotting, and quantified using ImageJ software.

### Biofilm formation assay

The wild type *L. monocytogenes* EGD (1 × 10^6^ CFU) with the indicated concentrations of atractylodin (0, 4, 8 μg/mL) and the mutant strain EGDΔ*hly* (1 × 10^6^ CFU) was added to a 24-well polystyrene microtiter plate. After 24 h-incubation, the crystal violet (CV) staining was operated as described in a previous study (16).

### Real-time quantitative PCR (RT-qPCR)

*L. monocytogenes* EGD was co-incubated with atractylodin (0, 4, 8 μg/mL) for 8 h and then total RNA of bacteria was extracted with TRIzol (Invitrogen). And the RNA was reverse-transcribed to cDNA using NovoScript^®^ Plus All-in-one 1st Strand cDNA Synthesis SuperMix (E047; Novoprotein, Shanghai, China). The levels of mRNA of *hly* in each sample were determined by real-time quantitative polymerase chain reaction (RT-qPCR) with NovoScript^®^SYBR qPCR SuperMix Plus (E096; Novoprotein, Shanghai, China). The data were collected and analyzed by the 2^−^ *^ΔΔCt^* method and normalized to 16sRNA. The mRNA level of the target genes in J774A.1 macrophages was detected by RT-qPCR as described above and GAPDH was used as an internal control. The primer pairs used for RT-qPCR are listed in **Supplementary Table 1**.

### Circular dichroism (CD) spectroscopy

LLO secondary structures in the presence or absence of atractylodin (4 μg/mL) were characterized using CD spectroscopy. Briefly, CD spectra were acquired by recording spectra at the scanning wavelengths ranging from 190-250 nm at a 0.5 nm interval using a Jasco J-810 spectrometer and analyzed *via* the BeStSel web server (https://bestsel.elte.hu/index.php).

### Molecular docking and molecular dynamics (MD) simulations

The structure of LLO (PDB ID: 4CDB) used in the current study was obtained from the RCSB Protein Data Bank. Then, the atractylodin-LLO complex molecule docking was performed using AutoDock Vina 1.1.2. MD simulations were performed using AMBER software (17) to assess the dynamic features of the atractylodin-LLO complex, whereby mimic the behavior in actual environment. In brief, MD simulation protocols consist of four major steps: minimization, heating, equilibration and production run. Force field selected for atractylodin is the general AMBER force field (GAFF) (18) and for LLO is ff14SB (19).

### Cytotoxicity and cytoprotection assay

Listeriolysin O is a crucial virulence factor to mediate vacuolar escape. The toxicity of atractylodin on J774A.1 cells and mouse peritoneal macrophages were all determined by using a Cell Counting Kit-8 (CCK-8; APExBIO) according to the manufacturer’s instructions. Additionally, J774A.1 macrophages were plated in 96-well-plates at a density of 3.0 × 10^4^ cells/well and grown overnight, then incubated with purified LLO protein or EGD (MOI=50) for 6 h at 37°C, with or without the indicated concentrations of atractylodin. Then, LDH released into the supernatants were determined with a Cytotoxicity Detection Kit (LDH; Roche, Basel, Switzerland). Furthermore, we analyzed the protective effect of atractylodin against LLO-mediated cells injury using the LIVE/DEAD (green/red) reagent (Invitrogen, USA).

### Intracellular growth assay

J774A.1 macrophages were seeded in 24-well-plates at a density of 2×10^5^ per well and cultured overnight. The cells were infected with EGD or EGDΔ*hly* at an MOI of 5 for 30 min with or without atractylodin (4 μg/mL). And then the cells were washed three times with sterile PBS and the remaining extracellular bacteria were killed with 50 µg/mL gentamicin, and simultaneously incubated with atractylodin (4 μg/mL).

At arranged time points (0.5 h, 2 h, 5 h), after thrice additional washes, the cells were lysed with sterile water to count the number of invasive bacteria by microbiological plating. Additionally, J774A.1 macrophages were infected with EGDΔ*hly*::*hly* in the same manner and then calculated the number of invasive bacteria as described above.

### Immunofluorescent staining

J774A.1 cells were seeded in 24-well-plates overnight and infected with *L. monocytogenes* EGD at an MOI of 2 plus the indicated concentrations of atractylodin (4 μg/mL). At 6 h post-infection (pi), infected cells were washed with PBS and then fixed with 4% paraformaldehyde for 30 minutes. After blocking with 5% BSA for 2 h, the cells were incubated with anti-Nrf2 antibody overnight and secondary antibody conjugated to Alexa Fluor 594 (Molecular Probes) for 1 h. After straining with DAPI for 7 min and washing with PBS, the images were obtained using an inverted fluorescence microscope (Olympus).

### Western blot analysis

J774A.1 cells were seeded on 24-well-plates overnight and infected with EGD or EGDΔ*hly* at an MOI of 2 in the presence or absence of atractylodin (4 μg/mL). After infected at 37°C for 6 h, the nuclear and cytoplasmic proteins were extracted using the Nuclear and Cytoplasmic Protein Extraction Kit (Beyotime, China) under the guidance of the instruction. And the cells were lysed by T-PER™ Tissue Protein Extraction Reagent (Thermo Fisher Scientific, USA) to obtain the total protein. In addition, the liver tissues of mice in histopathologic analysis were lysed by T-PER™ Tissue Protein Extraction Reagent to extract total protein. And then protein concentrations were quantified by the BCA method (Beyotime, China). The equivalent protein samples were respectively separated by 10% SDS-polyacrylamide gel electrophoresis (SDS-PAGE) and transferred onto polyvinylidene difluoride (PVDF) membranes. Blocked with 5% (w/v) nonfat milk for 2 h and the membranes were incubated with primary antibody at 4°C overnight. Then, the membranes were incubated with HRP-conjugated secondary antibody, and visualized with the ECL western blotting system. Additionally, the intensity of bands were quantified using ImageJ software.

### ROS measurement

J774A.1 cells plated in 24-well-plates were infected with *L. monocytogenes* EGD at MOI of 2, 5 or 10 in the presence or absence of atractylodin (4 μg/mL) for 1 h or 6 h. The culture medium was removed and then the adherent cells were washed with PBS, and incubated with 10 μM 2′,7′-dichlorofluorescein diacetate (DCFH-DA; Beyotime, China) for 30 min at 37°C. Subsequently, the cells were washed with PBS, removed and pelleted at 1500 r.p.m. for 5 min, resuspended in PBS and measured with the excitation wavelength at 488 nm and emission wavelength at 525 nm.

### The measurement of K^+^ efflux

J774A.1 macrophages in 24-well-plates were infected with *L. monocytogenes* EGD at an MOI of 2 with or without atractylodin (4 μg/mL) for 6 h. And intracellular K^+^ was measured using a Potassium Assay Kit under the guidance of the instruction.

### ELISA assay

For the detection of inflammatory cytokines (such as TNF-α and IL-6), mouse peritoneal macrophages were infected with *L. monocytogenes* EGD supplemented with or without atractylodin (4 μg/mL), as performed in cell infection assays and the cell supernatants were collected. Inflammatory cytokines in the cell supernatants or liver tissues in mice were respectively assessed using ELISA MAX™ Deluxe Set, strictly following the manufacturer’s instructions. Absorbance values were monitored using microplate reader at 450 nm and the concentrations were calculated according to the standard curves.

### Animal experiments

Exponentially growing *L. monocytogenes* EGD was collected, resuspended with sterile PBS buffer, followed by adjusting the bacteria density for different aims.

To analyze the protective effect of atractylodin *in vivo*, 100 µL prepared bacterial suspension (1 × 10^7^ CFU per mouse) was intraperitoneally inoculated into Balb/C mice and then randomly assigned. The certain concentration of atractylodin (60 mg/kg/day) was subcutaneous injected at 12-h intervals for 72 h in the treated group and the same volume of solvent DMSO was injected in the positive control group. The survival of infected mice was always monitored for the analysis of mortality studies.

For histopathologic analysis, Balb/C mice were intraperitoneally injected with *L. monocytogenes* EGD (5 × 10^6^ CFU per mouse) to establish systemic infection model and treated with or without atractylodin (30 mg/kg/day in low-dose group or 60 mg/kg/day in high-dose group) at 12-h intervals. The mice were euthanized at 48 h post-infection. Liver, spleen and kidney tissues were collected and lysed to transplant onto TSB solid medium to analyze the bacterial burden. For another, fix with 10% formaldehyde for the histopathology assessment and liver tissues of mice were collected and total protein was extracted for the western blotting assays as described above. In addition, to examine the level of inflammation in the mice liver tissues at 48 h post-infection, the TNF-α, IL-6, IL-1β and IFN-γ secretions in the livers were analyzed according to the manufacturer’s instructions.

### Measurement of ALT, AST, MPO and SOD levels in liver tissues

Analysis for alanine aminotransferase (ALT) and aspartate aminotransferase (AST) are vital to assess hepatic injury and malfunction accurately. Furthermore, myeloperoxidase (MPO) as a marker of the intensification of lipid peroxidation and the oxidative stress was assessed, and the anti-oxidative enzyme SOD activity was used to evaluate the antioxidant reserve.

All mice were euthanized at 48 h post-infection, and liver tissues were homogenized to analyze the ALT, AST, MPO and SOD levels using the respective assay kits and all procedures were performed according to the manufacturer’s instructions.

### Statistical analysis

All data were expressed as the means ± SEM and analyzed using GraphPad Prism 8.0 software. Unpaired two-tailed Student’s *t* test was employed for the comparison between two independent groups. Multiple comparisons between more than two groups were performed using one-way analysis of variance (ANOVA). And statistical significance was defined as *P* < 0.05.

## Results

### Atractylodin observably inhibits LLO function

**Figure 1:**
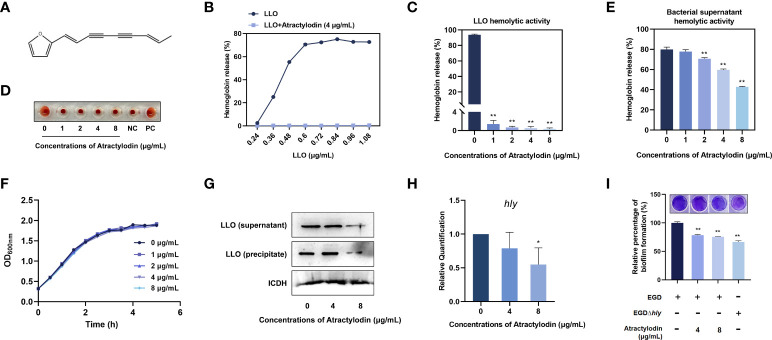
Atractylodin observably inhibited LLO function. **(A)** Chemical structure of atractylodin. **(B)** Inhibition of atractylodin on indicated concentrations of LLO-mediated hemolytic activity. Hemolytic activity of LLO **(C)** and the supernatants of *L. monocytogenes* EGD cultures **(E)** pre-incubated with or without atractylodin. **(D)** A corresponding visual image of inhibitory effect of atractylodin on LLO-mediated hemolytic activity. The hemolytic activity was represented as value obtained at OD_570nm_, and RBCs treated with ddH_2_O sample served as a positive-control (PC) and untreatment RBCs sample served as a negative-control (NC). **(F)** Growth curves for the *L. monocytogenes* EGD co-cultured with indicated concentrations of atractylodin. **(G)** Western blotting analysis of LLO expression in cultured supernatant and precipitate. ICDH was acted as an internal control. **(H)** Inhibition of the transcription of the genes *hly* encoding LLO by atractylodin was evaluated by RT-qPCR analysis. **(I)** Quantification of biofilm biomass by crystal violet (CV) staining. All of the data are representative of three independent experiments and presented as the mean ± SEM. *P < 0.05, **P < 0.01.

An important mechanism of *L. monocytogenes* dissemination involves cell-to-cell spread *via* the secretion of a plethora of virulence factors. Listeriolysin O (LLO) as a thiol-activated cholesterol-dependent pore-forming toxin contributes substantially to bacterial invasion and infection by disrupting the plasma membrane. In the present study, atractylodin (**Figure 1A**) was identified as an effective LLO inhibitor, as evidenced by the overwhelmingly decreased LLO-induced hemolysis (**Figure 1B**). Further, with the increasing of atractylodin concentrations, hemolytic activities induced by recombinant LLO (**Figures 1C, D**) and culture supernatants of *L. monocytogenes* EGD (**Figure 1E**) were prominently inhibited. And atractylodin administration exhibited no visible effect on *L. monocytogenes* EGD growth (**Figure 1F**). Additionally, the LLO expression in atractylodin‐treated *L. monocytogenes* was significantly decreased, in comparison with the control group in the absence of atractylodin treatment (**Figure 1G**). Consistent with these results, the production of LLO in *E. coli* BL21-pET21a-LLO induced by IPTG was also dramatically inhibited by atractylodin treatment **(** **Supplementary Figure 1A**), which may associate with the underlying mechanisms of specific LLO expression in a procaryotic system. Likewise, the decreased mRNA levels of *hly*, encoding LLO, in *L. monocytogenes* treated with atractylodin also add up to the consistency of result (**Figure 1H**).

Considerable evidence has emerged indicating that LLO as a pivotal effector contributes to *L. monocytogenes* biofilm formation (20). Thus, we further examined the effect of atractylodin on the formation of bacterial biofilm. As shown in **Figure 1I**, with the increasing concentrations of atractylodin, quantifying biofilm biomass by crystal violet (CV) staining was decreased, indicating that *L. monocytogenes* biofilm formation was significantly suppressed by atractylodin treatment.

Collectively, our results established that atractylodin thwarted LLO function by simultaneously diminishing LLO production and hemolytic activity without impacting *L. monocytogenes* viability.

### Atractylodin blocks LLO pore formation by engaging the LLO active residues

**Figure 2:**
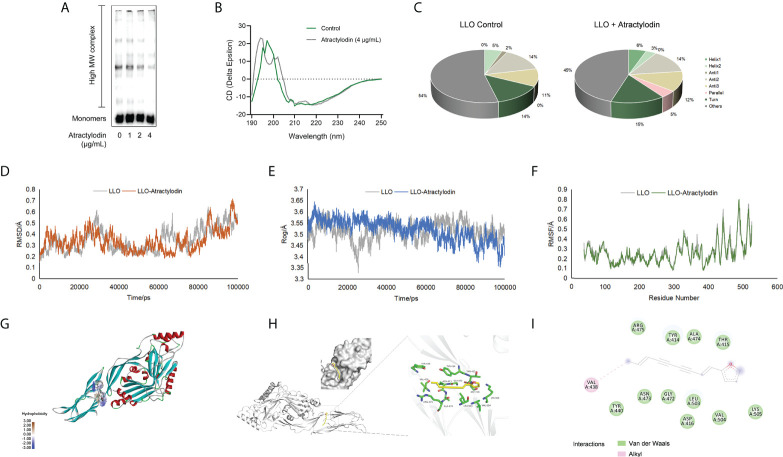
Molecular interactions between atractylodin and LLO structure. **(A)** The effect of atractylodin on LLO oligomerization was determined with an oligomer formation analysis. **(B, C)** The circular dichroism (CD) spectroscopy analysis of LLO in the presence or absence of 4 μg/mL atractylodin. The root mean square deviation (RMSD) **(D)**, radius of gyration (Rog) **(E)** and root mean square fluctuation (RMSF) **(F)** in free LLO protein and LLO-atractylodin complex were analyzed to determine the stability of the system involved in interactions between LLO and atractylodin. **(G, H)** The binding mode between atractylodin and LLO. **(I)** Interaction details and energy decomposition analysis of docked complex.

LLO, as a pore-forming toxins (PFTs), binds to the target membranes and subsequently oligomerizes to form functional pores *via* assembly of monomers into oligomeric structures on membranes, eventually lead to cell lysis (21). To elucidate the mechanisms underlying the anti hemolytic properties of atractylodin, the oligomerization assay was employed. As expected, LLO oligomers in atractylodin-treated samples were obviously reduced compared with the non-treated samples **(** **Figure 2A**, **Supplementary Figure 1B** **)**, suggesting that atractylodin interfered with the pore-forming activity by hindering LLO oligomerization.

To inspect and verify the molecular basis for the inhibition of LLO activity by atractylodin, circular dichroism (CD) spectra was employed to analyse the secondary structures of LLO in the presence of absence of atractylodin. Accordingly, atractylodin addition led to an obvious conformational change (**Figure 2B**), as evidenced by a significant increase in α-helix1 and decrease in α-helix2 (**Figure 2C**), suggesting that a direct engagement of atractylodin with LLO accompanying the alternation of second structure may contribute to the atractylodin-driven inhibition on LLO activity.

The potential LLO residues responsible for interacting with atractylodin were identified using molecular docking and molecular dynamics simulation to analyze the molecular interactions between atractylodin and LLO structure. As shown in **Figures 2D–F**, a production run of 100 ns was carried out and analyzed using root mean square deviation (RMSD), root mean square fluctuation (RMSF), and radius of gyration (Rog) to determine the stability of LLO/atractylodin complex. Overall spectrum of RMSD with the presence or absence of atractylodin showed no significant difference on structural shifts and RMSD value for the system was <1.0Å, indicating the stability of the LLO structure and strength of atractylodin attachment inside the active pocket (**Figure 2D**). Additionally, radius of gyration (Rog) was measured with or without atractylodin to calculate the structural compactness and equilibrium of the system. As depicted in **Figure 2E**, Rog with the presence of atractylodin showed an obviously diminishing tendency after 60 ns-simulation, suggesting a constrictive protein-structure unfavorable for assemble, which was congruent with LLO oligomerization result. RMSF as a crucial parameter reflects the structural flexibility in the current system, indicating that active residues appeared to be the potential binding sites to atractylodin (**Figure 2F**). The extension and contraction of helices were observed frequently during 0-100 ns, attributing to the presence of atractylodin inside LLO active site pocket. Collectively, the overall system exhibited appreciable stability as depicted in **Figures 2D–F**. Molecular docking analysis further showed potential interactions with atractylodin. Key active residues, such as VAL438, TYR440, ASN473, GLY472, LEU503, ASP416, VAL504, LYS505, THR415, ALA474, TYR414 and ARG475, were observed to be involved in atractylodin ligand attachment inside the active site pocket of LLO, hence depicting its inhibition efficacy (**Figures 2G, H**). Further, it was observed that the ligand is attached with the active site residue VAL438 with alkyl interaction, and residues TYR440, ASN473, GLY472, LEU503, ASP416, VAL504, LYS505, THR415, ALA474, TYR414 and ARG475 were involved in *van der Waals* interaction as shown in **Figure 2I**. These combined interactions formed by atractylodin interaction on LLO active sites illustrated its attachment and strength of interaction as required for the inhibition efficacy of atractylodin against LLO pore-forming function.

### Atractylodin remarkably alleviates *L. monocytogenes*-mediated cytotoxicity

**Figure 3:**
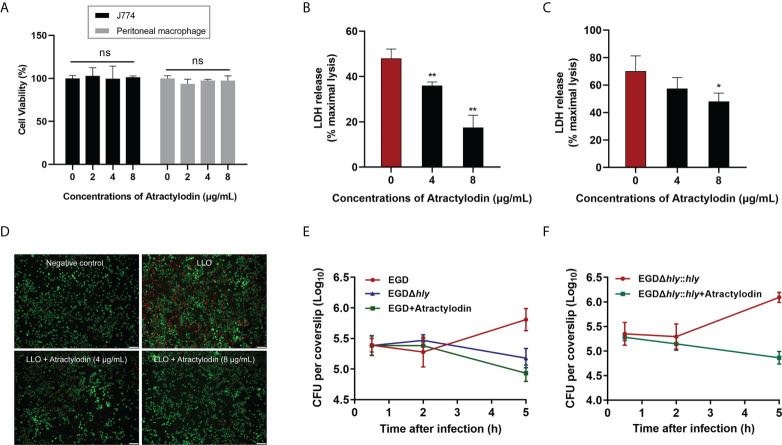
Atractylodin treatment ameliorated *L. monocytogenes* EGD‐induced cellular injury. **(A)** The cell toxicity determination of atractylodin on J774A.1 cells and peritoneal macrophages for 6 h using a CCK-8 kit. Cytotoxicity induced by LLO **(B)** and *L. monocytogenes* EGD **(C)** in J774A.1 cells with the presence and absence of atractylodin. **(D)** J774A.1 cells co-cultured with LLO in the presence of indicated concentrations of atractylodin were stained with the Live/Dead (green/red) reagent. Live cells were stained in green, and dead cells were stained in red. Scale bar, 50 μm. **(E)** Cells were infected with EGD in the presence or absence of atractylodin (4 μg/mL) or the *hly* deletion mutant EGDΔ*hly*. At 0.5, 2 and 5 h post-infection, cells were lysed and the total colony-forming units (CFUs) of intracellular bacteria were monitored by microbiology counting. **(F)** Intracellular growth curve of complementation strain EGDΔ*hly*::*hly* after co-incubated with or without atractylodin (4 μg/mL) were performed as described above. All of the data are representative of three independent experiments and presented as the mean ± SEM. ns, P > 0.05, *P < 0.05, **P < 0.01.

*L. monocytogenes* is a Gram-positive facultative intracellular pathogen, inducing cell death and tissue destruction. Notably, LLO is universally acknowledged as a precise tool for *L. monocytogenes* infection to modulate host cellular pathways in a manner that promotes infection (22). Herein, LLO or *L. monocytogenes* EGD mediated cellular viability in the presence or absence of atractylodin was assessed to determine whether atractylodin could confer cytoprotection. As depicted in **Figure 3A**, atractylodin, at the concentrations from 2 μg/mL to 8 μg/mL, displayed no evident cytotoxicity on host cells including J774A.1 cells and peritoneal macrophages. As expected, following incubation with LLO (**Figure 3B**) or *L. monocytogenes* EGD (**Figure 3C**) in J774A.1 cells, the released LDH in the atractylodin-treated samples were obviously decreased in comparison with the untreated cells (**Figures 3B, C**). Whereas, the comparatively impotent protective efficacy of atractylodin observed in *L. monocytogenes* EGD-challenged J774A.1 cells may attribute to the complex regulation strategies of *L. monocytogenes* causing cell death (23). Intuitively, exposed to atractylodin visibly alleviated LLO-induced cellular death, as evidenced by the decreased red fluorescence (**Figure 3D**). Based on this fact, to avoid the cytotoxicity of atractylodin, an effective and safe concentration, 4 μg/mL atractylodin was employed for further mechanisms exploration in cellular level.

To further explore the efficacy of atractylodin on the processes of *L. monocytogene* invading J774A.1 cells, we tested the intracellular bacterial growth. As shown in **Figure 3E**, the CFU burden in atractylodin-treated samples, as well as the samples with EGDΔ*hly* infection, were significantly decreased than untreated control at 5 h post-infection (pi). Notably, atractylodin-induced inhibition was also observed in the complementation strain EGDΔ*hly*::*hly-*infected cells (**Figure 3F**). Hence, our data suggested that atractylodin treatment could effectively restrain the replication of *L. monocytogenes* in host cells and protect against *L. monocytogene*-induced cellular injury.

### Atractylodin effectively attenuates *L. monocytogenes* pathogenicity *in vivo*

**Figure 4:**
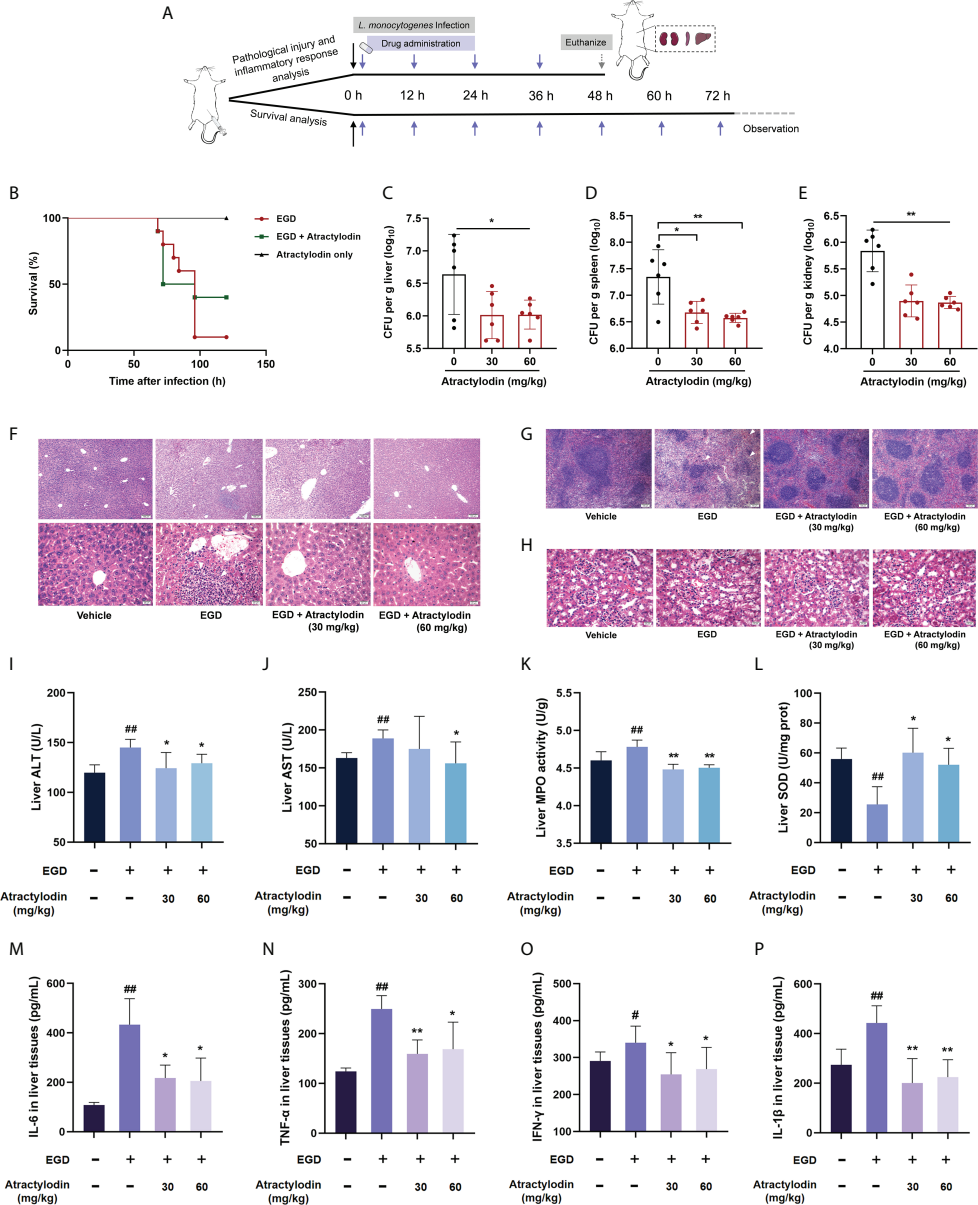
Atractylodin treatment protected mice from *L. monocytogenes* infection. **(A)** Scheme of the experimental protocols for *L. monocytogenes* systemic infection model. Mice were infected with *L. monocytogenes* EGD intraperitoneally and received a hypodermic injection of atractylodin (30 or 60 mg/kg/day) at 12-h intervals. **(B)** Survival analysis of mice treated with solvent control or 60 mg/kg/day atractylodin (n = 10 per group). The bacterial burden in the livers **(C)**, spleens **(D)** and kidneys **(E)** were determined at 48 h post-infection in the control group and the atractylodin-treated groups (n=6 per group). Histopathological observations of livers **(F)**, spleens **(G)** and kidneys **(H)** by H&E staining. The typical lesions in *L. monocytogenes* EGD infected mice were marked with white triangle. **(I–L)** Effects of atractylodin on levels of ALT, AST, MPO and SOD from liver homogenates of EGD-infection mice. Cytokines levels of IL-6 **(M)**, TNF-α **(N)**, IFN-γ **(O)** and IL-1β **(P)** in liver homogenates were assessed using ELISA. The data are presented as the mean ± SEM. ^#^ *P* < 0.05 and ^##^ *P* < 0.01 vs the vehicle control group; *P < 0.05 and **P < 0.01 vs EGD-infection group.

To further assess the protective potential of atractylodin against *L. monocytogenes* infection *in vivo*, the mice systemic infection model was established as depicted in **Figure 4A**. In agreement with *in vitro* cytotoxicity assays in **Figure 3A**, no death was observed within 120 h for the mice received subcutaneous injection of atractylodin at 60 mg/kg/day (**Figure 4B**). As expected, the infected mice received atractylodin (60 mg/kg/day) exhibited a significantly higher survival rate than the control group treated with DMSO, as evidenced by 40% survival vs 10% survival, indicating that atractylodin treatment evidently protected *L. monocytogenes*-infected mice from lethal infection (**Figure 4B**). Congruently, the bacterial burden in liver (**Figure 4C**), spleen (**Figure 4D**) and kidney (**Figure 4E**) of atractylodin-treated mice were substantially diminished. Dramatically, severe cellular degeneration and hemorrhage, congestion, inflammatory cell infiltration in livers (**Figure 4F**), necrosis with congestion in germinal centers of spleens (**Figure 4G**), prominent tubular dilatation and renal tissue integrity damage (**Figure 4H**) were observed in *L. monocytogenes*-infected mice, whereas which were obviously alleviated by atractylodin therapy (**Figures 4F–H**).

To further assess the therapeutic effects of atractylodin against *L. monocytogenes* infection, the AST and ALT levels in livers, key markers of hepatic injury, were evaluated. As presented in the **Figures 4I**, **J**, atractylodin treatment could significantly inhibit ALT and AST levels induced by *L. monocytogenes* EGD infection. Additionally, atractylodin treatment could obviously decrease MPO level (**Figure 4K**), one of the most important marker of oxidative stress and tissue damage, and simultaneously increase SOD content in the livers of mice infected with *L. monocytogenes* (**Figure 4L**). Thus, these findings suggested that atractylodin exhibited a potential anti-oxidation and anti-inflammatory properties against *L. monocytogenes* infection *in vivo.*

Consistent with the pathological analysis, *L. monocytogenes* infection markedly enhanced the level of IFN-γ, TNF-α, IL-6, and IL-1β in liver tissues, by contrast, which were prominently decreased by atractylodin treatment (**Figures 4M–P**), suggesting that atractylodin has potent anti-inflammatory effects against *L. monocytogenes* infection. Taken together, our results emphasized that atractylodin display promising therapeutic potential against *L. monocytogenes* infection by both suppressing LLO function and modulating inflammation response.

### Atractylodin enhances Nrf2/HO-1 signaling pathway and downregulates *L. monocytogenes*-activated NLRP3 inflammasome in mice

**Figure 5:**
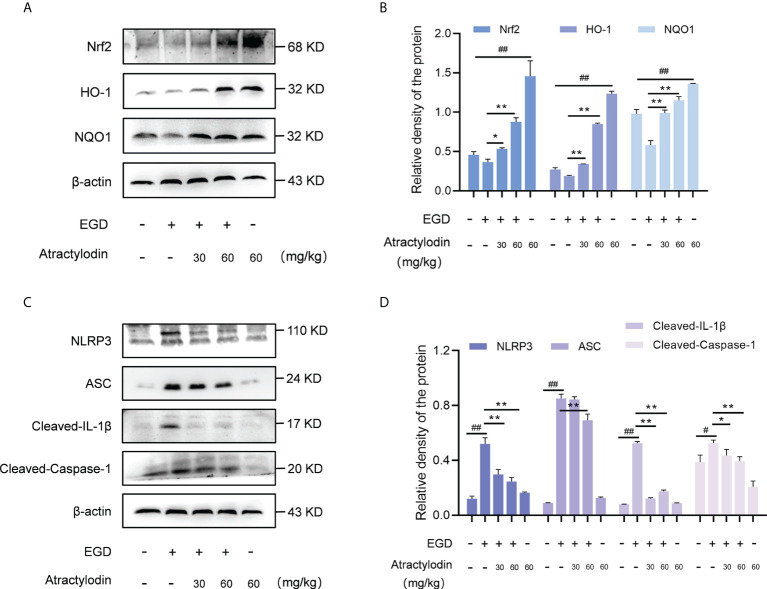
Effects of atractylodin treatment on Nrf2/NLRP3 signaling pathways in *L. monocytogenes* EGD-infection mice. At 48 h post-infection, liver tissues of mice were collected and analyzed by western blotting. **(A, B)** Effects of atractylodin on Nrf2, HO-1 and NQO1 protein expression in the livers. **(C, D)** Western blot analysis of NLRP3 inflammasome activation induced by *L. monocytogenes* infection in the presence of indicated concentrations of atractylodin. **(B, D)** Quantification of relative protein was determined by densitometric analysis. β-actin was served as an internal control. All data were expressed by mean ± SEM. ^#^ *P* < 0.05 and ^##^ *P* < 0.01 vs the vehicle control group; *P < 0.05 and **P < 0.01 vs EGD-infection group.

Recently, Nrf2 signaling pathway has attracted increasing interest in diverse bacterial infection; hence, the application of Nrf2 inducers might represent a novel therapy against pathogens infection (7). Notably, in light of the critical role of Nrf2 in NLRP3 inflammasome activation, Nrf2/NLRP3 signaling pathway was identified as regulators of multiple inflammatory diseases (24). Based on this fact, we were prompted to investigate the underlying mechanism of anti-inflammatory properties of atractylodin against *L. monocytogenes* infection in mice. Atractylodin treatment obviously enhanced Nrf2 activation (**Figures 5A, B**). Further, exposed to different doses of atractylodin increased the level of ARE genes, including HO-1 and NQO1 as depicted in **Figures 5A**, **B**, indicating that atractylodin could effectively activate Nrf2-mediated antioxidant defense in mice.

As previously reported (25, 26), *L. monocytogenes* infection manipulates the host inflammation response by NLRP3 inflammasome activation in mice. Consistent with this, *L. monocytogenes* EGD infection caused pronounced activation of NLRP3 inflammasome, whereas which was significantly attenuated by atractylodin treatment (**Figures 5C, D**). Subsequently, NLRP3-mediated caspase-1 activation and IL-1β maturation were inhibited by atractylodin and apoptosis-associated speck-like protein containing CARD (ASC) level was visibly decreased in the samples with atractylodin treatment (**Figures 5C, D**). Together, our results suggested that atractylodin possesses anti-inflammatory potentials for fighting *L. monocytogenes* infection by upregulating Nrf2 pathway and inhibiting NLRP3 inflammasome activation in mice.

### Atractylodin prompts Nrf2 translocation into nucleus, independent of LLO, to regulate Nrf2/NLRP3 signaling pathway in macrophages

**Figure 6:**
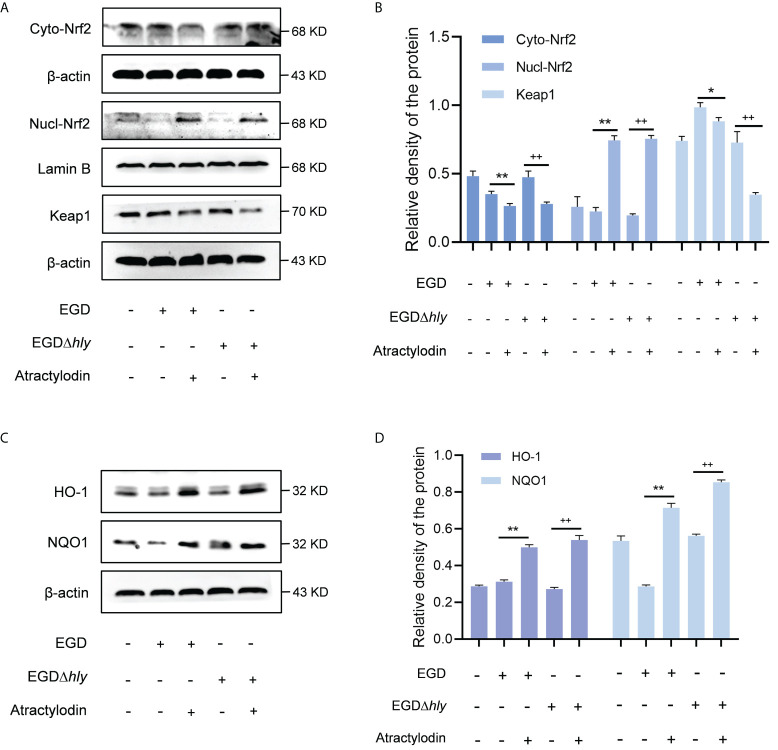
Atractylodin facilitated the nuclear translocation of Nrf2, thereby upregulated the antioxidant enzyme expression in *L. monocytogenes* infected macrophages. **(A, B)** Western blot analysis of effects of atractylodin on the Keap1-Nrf2 signaling pathway during *L. monocytogenes* infection. Nuclear and cytoplasmic protein from J774A.1 cells were prepared and analyzed by western blotting for the assessment of the nuclear translocation of Nrf2. **(C, D)** The levels of Nrf2-targeted typical phase 2 detoxifying enzyme NQO1 and the stress response protein HO-1 in J774A.1 cells infected with wildtype *L. monocytogenes* strain EGD and the *hly*-deficient mutant EGDΔ*hly* in the presence or absence of atractylodin. **(B, D)** The levels of Cyto-Nrf2, Keap1, NQO1 and HO-1 relative to β-actin and Nucl-Nrf2 relative to Lamin B determined using densitometry were shown. Data are represented as the mean ± SEM. *P < 0.05 and **P < 0.01 vs the EGD-infection group; ^++^ *P* < 0.01 vs the EGDΔ*hly*-infection group.

Nrf2, as a key redox-sensitive transcription factor responses for the progression of bacterial infection (27, 28), by modulating diverse phase 2 detoxifying enzymes and various stress-responsive proteins. Under unstressed conditions, the interaction between Nrf2 and Keap1 causes continual ubiquitination and degradation of Nrf2 in the cytoplasm. Once cellular stress occurs, Nrf2 is accumulated and translocated to the nucleus, binding to the antioxidant response element (ARE) to launch the antioxidant defense (29). Given that the complex regulatory mechanisms of Nrf2, studies on J774A.1 macrophages revealed that atractylodin observably prompted the nuclear translocation of Nrf2 during *L. monocytogenes* infection, as evidenced by an increase in the nuclear levels of Nrf2 and an accompanied decrease in the cytoplasmic levels of Nrf2 (**Figures 6A, B**). As indicated in **Supplementary Figure 2A**, Lamin B nuclear marker was employed to verify the samples with no cross contamination between nuclear and cytoplasmic fractions. Consistent with these observations, immunofluorescence analysis confirmed that following the treatment of atractylodin, visibly more Nrf2 located in the nucleus of the *L. monocytogenes* EGD infected macrophages (**Supplementary Figure 2B**). Additionally, atractylodin-driven Keap1 degradation in *L. monocytogenes* infected host cells further highlighted the pivotal role of atractylodin on Keap1-Nrf2 system (**Figures 6A, B**). Accordingly, the addition of atractylodin (4 μg/mL) visibly enhanced the level of Nrf2-targeted ARE genes, including NQO1 and HO-1, in cells infected by EGD and EGDΔ*hly* (**Figures 6C, D**), suggesting that LLO may not be involved in atractylodin-induced Nrf2 activation during *L. monocytogenes* infection. Intriguingly, such increases in the Nrf2-Keap1 system were also observed in uninfected macrophages with atractylodin treatment (**Supplementary Figures 3A, B**). Previous evidences identified that ROS was critically involved in the mechanism of Nrf2 activation (30). Therefore, we assessed the effects of atractylodin on *L. monocytogenes* evoked ROS production under different stimulation conditions. As depicted in **Supplementary Figures 2C**, **D**, atractylodin addition (4 μg/mL) significantly diminished ROS level at 6 h post-infection, rather at 1 h pi. Together, these results concluded that atractylodin treatment could markedly upregulate the Keap1-Nrf2/ARE signaling pathway, independent of LLO, to display an antioxidant potential in *L. monocytogenes*-infected macrophages.

**Figure 7:**
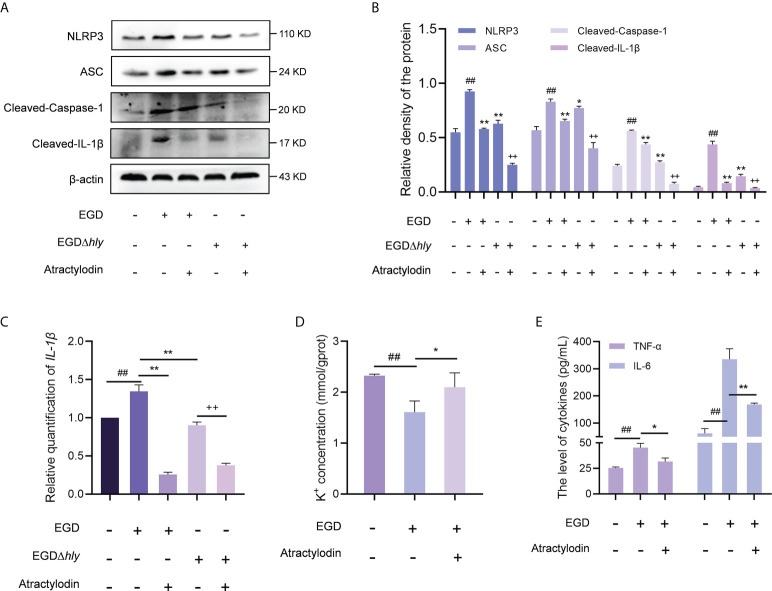
Atractylodin effectively suppressed *L. monocytogenes*-activated NLRP3 inflammasome. **(A)** J774A.1 cells were infected with EGD or EGDΔ*hly*, following the treatment with atractylodin (4 μg/mL) for 6 h and then immunoblotting with specific antibodies. **(B)** The levels of NLRP3, ASC, Cleaved-Caspase-1 and Cleaved-IL-1β relative to β-actin determined using densitometry were shown. **(C)** The relative expression of mRNA of *IL-1β* in J774A.1 cells in the presence or absence of atractylodin was determined by RT-qPCR. **(D)** The intracellular content of K^+^ in J774A.1 cells with the presence of DMSO or 4 μg/mL atractylodin was evaluated using a K^+^ detection assay kit. **(E)** The production of IL-6 and TNF-α in the peritoneal macrophage treated with or without atractylodin were measured using ELISA. Data are represented as the mean ± SEM. ^##^ *P* < 0.01 vs the vehicle control group; *P < 0.05 and **P < 0.01 vs the EGD-infection group; ^++^ *P* < 0.01 vs the EGDΔ*hly*-infection group.

In line with the *in vivo* analysis, the NLRP3 inflammasome was significantly activated in *L. monocytogenes* infected macrophages with an enhanced expression of cleaved caspase-1, ASC and IL-1β, whereas which were observably inhibited by atractylodin treatment in EGD or EGDΔ*hly* infected macrophages (**Figures 7A, B**), suggesting an LLO independent anti-inflammation mechanism of atractylodin against *L. monocytogenes* infection. Additionally, atractylodin treatment exhibited no obvious effect on NLRP3 signaling pathway in the uninfected cells (**Supplementary Figures 3C, D**). Notably, NLRP3 inflammasome was also decreased in the EGDΔ*hly* infected samples (**Figures 7A, B**), indicating that LLO is required for the activation of NLRP3 inflammasome during *L. monocytogenes* infection. Congruently, atractylodin treatment remarkably attenuated *L. monocytogenes*-induced IL-1β expression at both the protein (**Figures 7A, B**) and mRNA levels (**Figure 7C**). As consequently, a dramatical inhibition of K^+^ efflux responses, the NLRP3 inflammasome signaling downstream, was also observed by atractylodin addition (**Figure 7D**). Consistently, TNF-α and IL-6 secretion in the *L. monocytogenes* infected mouse peritoneal macrophages were detected to further evaluate the efficacy of atractylodin on host-derived inflammation. As shown in **Figure 7E**, atractylodin thwarted the productions of IL-6 and TNF-α in EGD-infected macrophages, suggesting that the suppression of NLRP3 inflammasome activation by atractylodin led to a decreased inflammation response in *L. monocytogenes* infected host cells. Taken together, our results indicated that atractylodin treatment effectively suppressed *L. monocytogenes*-evoked inflammation response and enlightened that the coordinated regulation of Nrf2 and NLRP3 may play a crucial role in the therapeutic effects of atractylodin against *L. monocytogenes* infection.

### Regulation of Nrf2/NLRP3 signaling pathway by atractylodin is dependent on Nrf2

**Figure 8:**
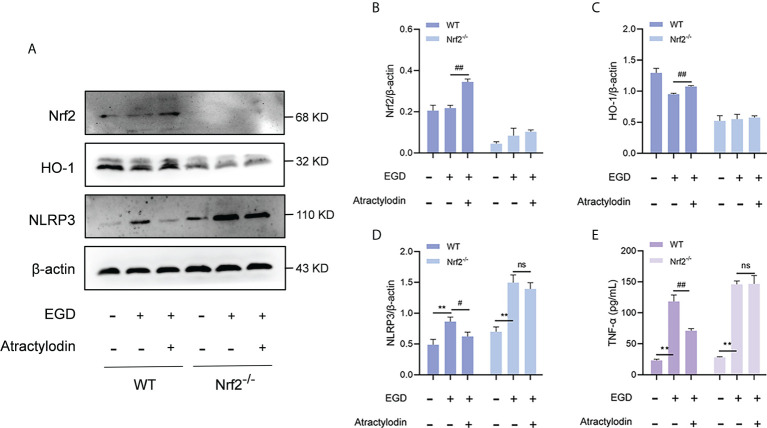
The inhibitory effect of atractylodin on NLRP3 inflammasome was dependent upon Nrf2 activation. Peritoneal macrophages from wide-type (WT) and Nrf2 knock out (Nrf2^-/-^) mice were isolated and infected with *L. monocytogenes* EGD for 6 h in the presence or absence of atractylodin. **(A–D)** Representative immunoblots and quantification of Nrf2, HO-1, NLRP3 protein levels in WT and Nrf2^-/-^ mice. **(E)** Effects of atractylodin on the level of TNF-α secretion in the co-cultured supernatants. All data are presented as means ± SEM. **P < 0.01 vs the vehicle control group; ns, *P* > 0.05, ^#^ *P* < 0.05 and ^##^ *P* < 0.01 vs the EGD-infection group.

Accumulating evidences demonstrate that a negative regulatory role of Nrf2 on NLRP3 inflammasome activation (24). To investigate whether the inhibitory effect of atractylodin on NLRP3 inflammasome activation was mediated by Nrf2, peritoneal macrophages from wild-type mice and Nrf2^-/-^ mice were employed. As shown in **Figures 8A–D**, atractylodin treatment significantly inhibited *L. monocytogenes-*induced upregulation of NLRP3 in wild-type mice. However, the inhibitory effect of atractylodin on NLRP3 inflammasome activation was abolished in macrophages from Nrf2^-/-^ mice (**Figures 8A–D**). Consistent with these data, atractylodin mediated decrease of TNF-α production was observed in macrophages of the wild-type mice, rather in Nrf2^-/-^ mice (**Figure 8E**). Collectively, our results established that Nrf2 is required for the regulation of Nrf2/NLRP3 signaling pathway by atractylodin.

## Discussion

The ever-increasing incidence of drug-resistant *L. monocytogenes* infections introduces a tremendous economic burden and seriously threatens the convenient therapeutic options (31); notably, the horizontal spread of resistance genes to *L. monocytogenes* is accelerating to overwhelm the health system worldwide, prompting the discovery and development of novel alternative therapeutic approaches. Given that *L. monocytogenes* utilizes a plethora of complex regulation mechanisms to promptly adapt to and thrive in divergent physiological contexts, focusing on non-lethal candidates targeting bacterial virulence and adjunctive host-directed therapies (HDT) is invigorated. Our work presented a promising strategy to tackle drug-resistant *L. monocytogenes* by developing a non-lethal drug candidate, synchronously modulating the bacterial virulence and the immune response of *L. monocytogenes*-host interactions.

Listeriolysin O (LLO) as a cholesterol-dependent pore-forming toxin substantially contributes to *L. monocytogenes* pathogenesis and host innate immune responses to infection, thus was presented to be a promising and pertinent therapy target. As our understanding of LLO function expands, LLO as a versatile tool allows *L. monocytogenes* to induce the plasma membrane disruption (32) and trigger inflammasome activation (33). In the current study, atractylodin was identified as a potential therapeutic candidate against *L. monocytogenes* infection by potently diminishing LLO pore-forming activity through different mechanisms. The combined interactions formed by atractylodin interaction on LLO active residues (VAL438, TYR440, ASN473, GLY472, LEU503, ASP416, VAL504, LYS505, THR415, ALA474, TYR414 and ARG475) illustrated its attachment and strength of interaction as required for atractylodin-driven LLO inhibition. LLO punctures the cytomembrane by assembly of monomers into oligomeric structures in a cholesterol- and time-dependent manner (34). Consistently, our results established that atractylodin efficiently blocked the pore-forming activity of LLO at the oligomerization stage and simultaneously thwarted bacterial intracellular proliferation, consequently significantly ameliorated LLO- or *L. monocytogenes-*induced cytotoxicity.

LLO is indispensable for *L. monocytogenes* pathogenesis, and inflammasome activation during *L. monocytogenes* infection, which is effectively rely on an LLO-mediated phagosomal disruption, manifesting as the significant impairment of IL-1β production and NLRP3 inflammasome activation by *L. monocytogenes* mutants lacking LLO (9). Consistent with these results, in comparison with wild-type *L. monocytogenes* EGD, NLRP3 inflammasome activation was observably suppressed following EGDΔ*hly* infection. Herein, to further investigate whether LLO mediated the regulation of NLRP3 signaling pathway by atractylodin, we utilized wild-type *L. monocytogenes* EGD and its *hly*-deficient mutant EGDΔ*hly* to infect the J774A.1 macrophages in the presence or absence of atractylodin. Interestingly, our results revealed that atractylodin-driven inhibition on *L. monocytogenes-*activated NLRP3 inflammasome was not absolutely mediated by LLO. Atractylodin-induced accumulation of intracellular K^+^ blocked NLRP3 inflammasome activation, providing new insight into the molecular mechanism of the anti-inflammation activity of atractylodin and broadened the applications of atractylodin in inflammatory diseases.

Nrf2 as a crucial transcription factor regulates cellular oxidative stress responses and modulates intracellular redox homeostasis. Evidences are accumulating that Nrf2 signaling pathway has attracted increasing interest in diverse bacterial infection; hence, the application of Nrf2 activators might represent a novel therapeutic strategy against pathogens infection (7). In the present study, we investigated the efficacies and mechanisms of atractylodin on the *L. monocytogenes*-induced oxidative stress response, regulated by Nrf2. Atractylodin impaired the ability of Keap1 to target Nrf2 for degradation, promote newly synthesized Nrf2 translocation to nucleus, and induce ARE-driven cytoprotective gene expression, such as HO-1 and NQO1 catalyze diverse detoxification reactions during *L. monocytogenes* infection. Moving forward, illustrating the mechanisms underlying the elevated activity on the Nrf2/HO-1 axis induced by atractylodin contributing to the mitigation of oxidative stress may provide evidences for the application of Nrf2 activators in *L. monocytogenes* infections. To prevent oxidative stress, the cells further respond to ROS by complex antioxidant defense systems. *L. monocytogenes*-triggered ROS generation was substantially diminished by atractylodin treatment at 6 h post-infection, suggesting atractylodin mediated the protection against *L. monocytogenes*-stimulated oxidative damage. Intriguingly, atractylodin positively regulated ROS production at 1 h post-infection, might attributing to the a distinct defense mechanism against pathogens *via* promoting ROS generation in phagocytes and subsequently activating the antioxidant transcription factor Nrf2 to maintain redox homeostasis in macrophages (35). In addition, atractylodin exhibited antioxidant and anti-inflammation effects *in vivo/in vitro* by upregulating the Nrf2/HO-1 signaling pathway to inhibit NLRP3 inflammasome activation. Overall, further in-depth molecular mechanisms exploration about the interaction between Nrf2 and the NLRP3 inflammasome would provide mechanistic insights and present a novel target against bacterial infections.

*Listeria monocytogenes*, as an ideal model pathogen, the pathogenic molecular mechanisms are complex and are generally considered as the result of host-pathogen coevolution. Thus, targeting the bacterial virulence and the host defense is a promising strategy for the development of anti-infection candidates. In this way, our findings illustrated the therapeutical effects and underlying mechanisms of atractylodin against *L. monocytogenes* infections *in vivo/in vitro.* Our work provided a novel lead compound and a promising therapeutic strategy to combat *L. monocytogenes* infections, which is of practical significance for clinical therapeutics.

## Conclusions

**Figure 9:**
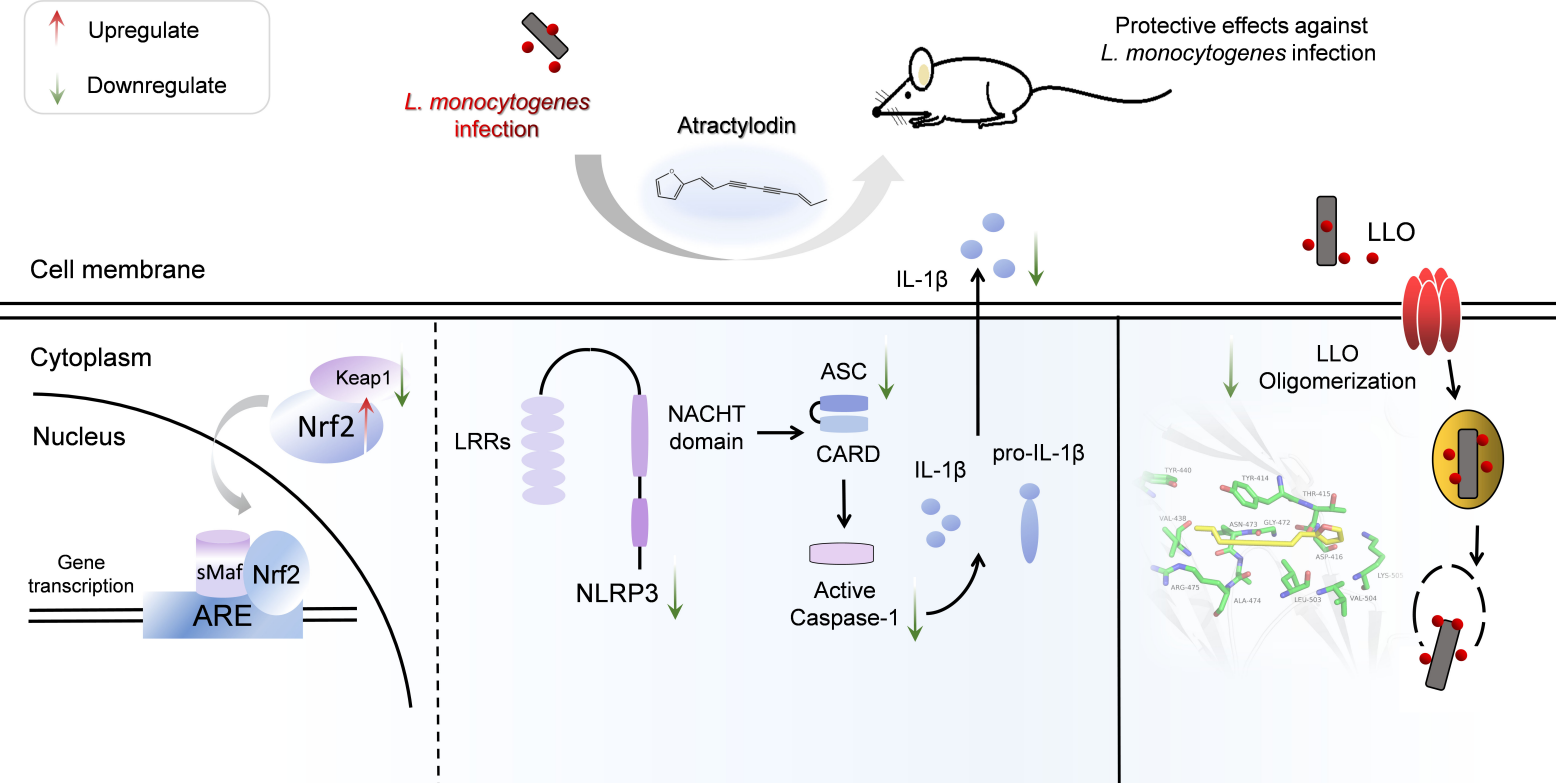
Scheme summarizing the protective effects of atractylodin against *L. monocytogenes* infection by targeting listeriolysin O and mediating the suppression of inflammation. Atractylodin, as a potent antagonist of LLO, effectively protected mice from *L. monocytogenes* infection against inflammation response which largely rely on upregulation of the Nrf2-mediated anti-oxidant pathways and inhibition of NLRP3 inflammation activation.

In summary, as illustrated in **Figure 9**, our research firstly indicated that atractylodin dramatically suppressed the pathophysiological process of *L. monocytogenes* by abolishing the LLO pore formation and effectively protected *L. monocytogenes*-challenged tissues injury against oxidative stress and inflammation damage, dependent on Nrf2 activation, and the inhibition of *L. monocytogenes*-activated NLRP3 inflammasome. Our study provides compelling evidences for the potential application of atractylodin in the prophylaxis and treatment of *L. monocytogenes* infection.

## Data availability statement

The raw data supporting the conclusions of this article will be made available by the authors, without undue reservation.

## Ethics statement

The animal study was reviewed and approved by the Animal Welfare and Research Ethics Committee in Jilin University.

## Author contributions

LX, YZ, XD, HF, and JW contributed to the conception of the experiments; LX, YZ, GL, JX, XX, QL, LW, and XS performed the experiments; LX, YZ, HF, and JW contributed conspicuously to analysis and manuscript preparation; LX, YZ, HF, and JW drafted this manuscript. All authors reviewed, revised, and approved the final report.

## Acknowledgments

The *L. monocytogenes* strains used in this study were given by Dr. Masao Mitsuyama (Kyoto University Graduate School of Medicine, Japan) as gifts. This work was supported by the National Natural Science Foundation of China (grant 81861138046, No. 31772798, No.31970507 and No. 31902321).

## Conflict of interest

The authors declare that the research was conducted in the absence of any commercial or financial relationships that could be construed as a potential conflict of interest.

## Publisher’s note

All claims expressed in this article are solely those of the authors and do not necessarily represent those of their affiliated organizations, or those of the publisher, the editors and the reviewers. Any product that may be evaluated in this article, or claim that may be made by its manufacturer, is not guaranteed or endorsed by the publisher.
