# Supplementary material for: Anti-inflammatory, antioxidant and anti-virulence roles of atractylodin in attenuating Listeria monocytogenes infection
Source: Front Immunol. 2022 Oct 28;13:977051. doi: 10.3389/fimmu.2022.977051 (PMC9651212; doi:10.3389/fimmu.2022.977051)
Supplement: Supplementary file 1 [file DataSheet_1.doc]

**Supplementary Table 1 Sequence of primers used for RT-qPCR assay.**

| **Gene** | **Primer** | | **Sequence (5´-3´)** |
| --- | --- | --- | --- |
| **Mice *IL-1β*** | Forward | | ACCTGTGTCTTTCCCGTGG |
|  | Reverse | | TCATCTCGGAGCCTGTAGTG |
| **Mice *GAPDH*** | Forward | | AGGTCGGTGTGAACGGATTTG |
|  | Reverse | GGGGTCGTTGATGGCAACA | |
| ***hly*** | Forward | TGCCAGGTAACGCGAGAAAT | |
|  | Reverse | TGGTGCCCCAGATGGAGATA | |
| ***16S rRNA*** | Forward | ACAGAACTAGGCGCACACAA | |
|  | Reverse | TTCACTAATGCCGCCCTCTG | |

**Abbreviations**

| **ALT** | liver alanine transaminase |
| --- | --- |
| **ASC** | apoptosis-associated speck-like protein containing CARD |
| **AST** | aspartate aminotransferase |
| **BSA** | bovine serum albumin |
| **CCK-8** | Cell Counting Kit-8 |
| **CDC** | cholesterol-dependent cytolysin |
| **DMSO** | Dimethyl Sulfoxide |
| **ELISA** | enzyme-linked immunosorbent assay |
| **GCLC** | glutamate-cysteine ligase catalytic subunit |
| **GCLM** | glutamate-cysteine ligase modifier subunit |
| **GPx** | glutathione peroxidase |
| **HO-1** | heme oxygenase-1 |
| **ICDH** | isocitrate dehydrogenase |
| **IL-6** | interleukin-6 |
| **LDH** | lactate dehydrogenase |
| **LLO** | Listeriolysin O |
| ***L. monocytogenes*** | *Listeria monocytogenes* |
| **MAPKs** | mitogen-activated protein kinases |
| **MD** | Molecular dynamics |
| **MOI** | multiplicity of infection |
| **MPO** | myeloperoxidase |
| **NLRP3** | NOD-, LRR- and pyrin domain-containing protein 3 |
| **Nrf2** | Nuclear factor erythroid 2-related factor 2 |
| **NQO1** | NADP(H):quinone oxidoreductase-1 |
| **pi** | post-infection |
| **RMSD** | root mean square deviation |
| **RMSF** | root mean square fluctuation |
| **Rog** | radius of gyration |
| **RT-qPCR** | Real-time quantitative PCR |
| **SOD** | superoxide dismutase |
| **TLR4** | toll-like receptor 4 |

[**Supplementary**](javascript:;) **figures and figure legends**

**
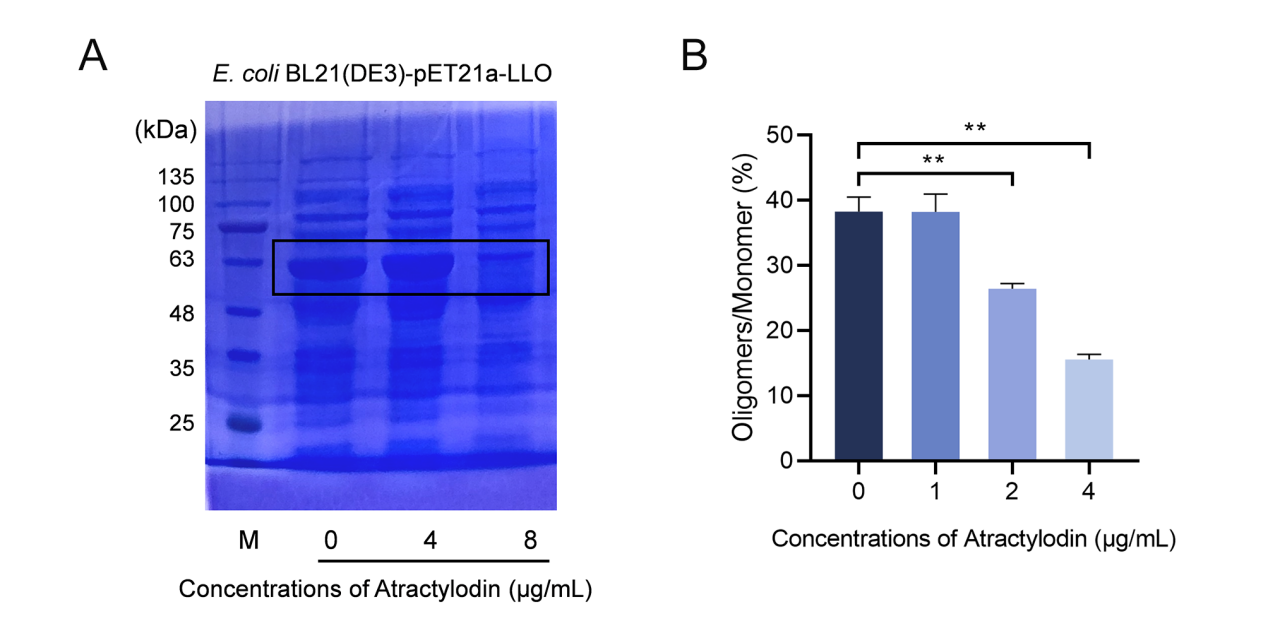
**

**Supplementary Figure 1 The underlying mechanisms of atractylodin-induced inhibitory effect on hemolysis. (A)** Atractylodin addition decreased the expression of LLO in *E.coli* BL21(DE3)-pET21a-LLO induced by IPTG. Samples were loaded on SDS-PAGE gels and analyzed by staining with Coomassie brilliant blue. **(B)** The optical density of LLO Oligomers/Monomer related to **Figure 2A** was analyzed with ImageJ software. All data are presented as means ± SEM. ***P* < 0.01.

**
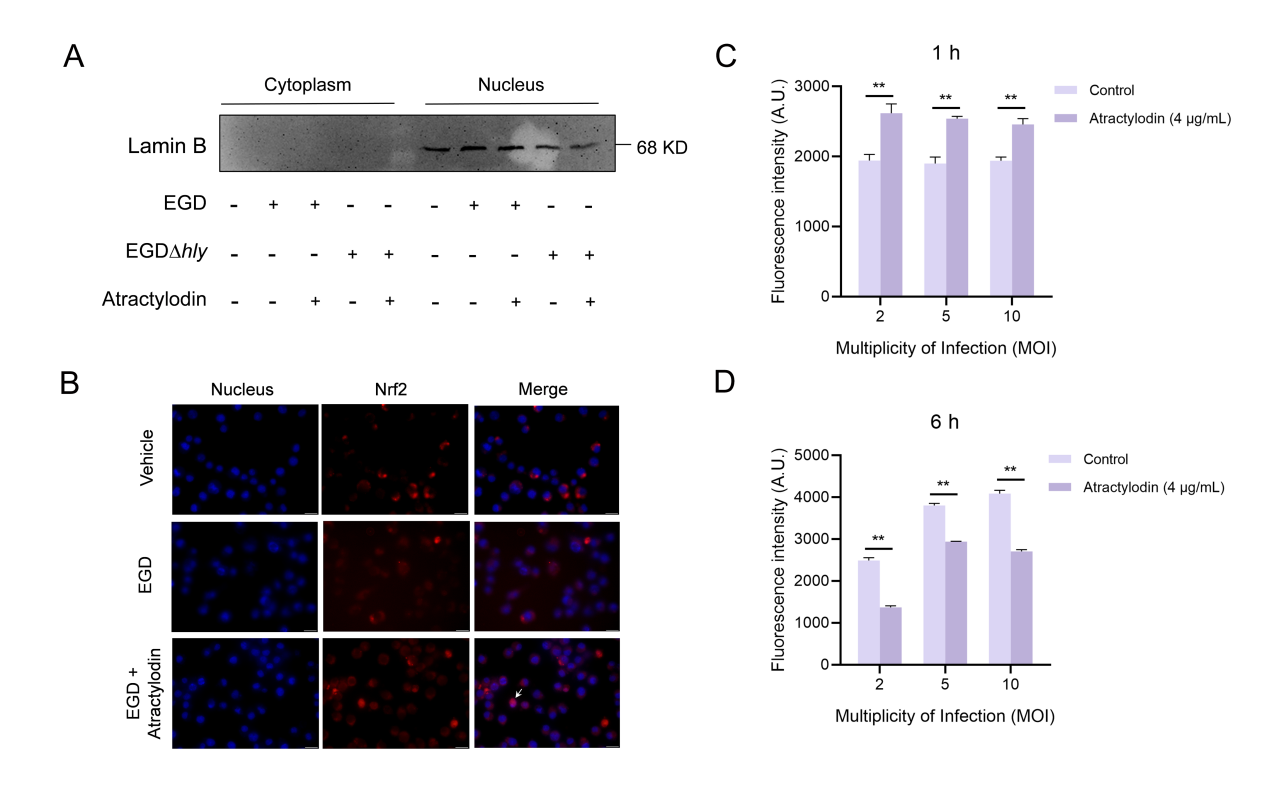
**

**Supplementary Figure 2 The effects of atractylodin on Nrf2 nuclear translocation and ROS generation during** ***L. monocytogenes* infection. (A)** Immunoblotting results for nuclear markers (Lamin B) were shown. **(B)** Immunofluorescence analysis was employed to evaluate the effects of atractylodin on Nrf2 nuclear translocation and expression. Scale bars, 2 μm. ROS level in J774A.1 cells infected with *L. monocytogenes* (MOI = 2, 5, 10) in the presence or absence of atractylodin (4 μg/mL) at 1 h **(C)** or 6 h **(D)** post-infection. All data are presented as means ± SEM. ***P* < 0.01.


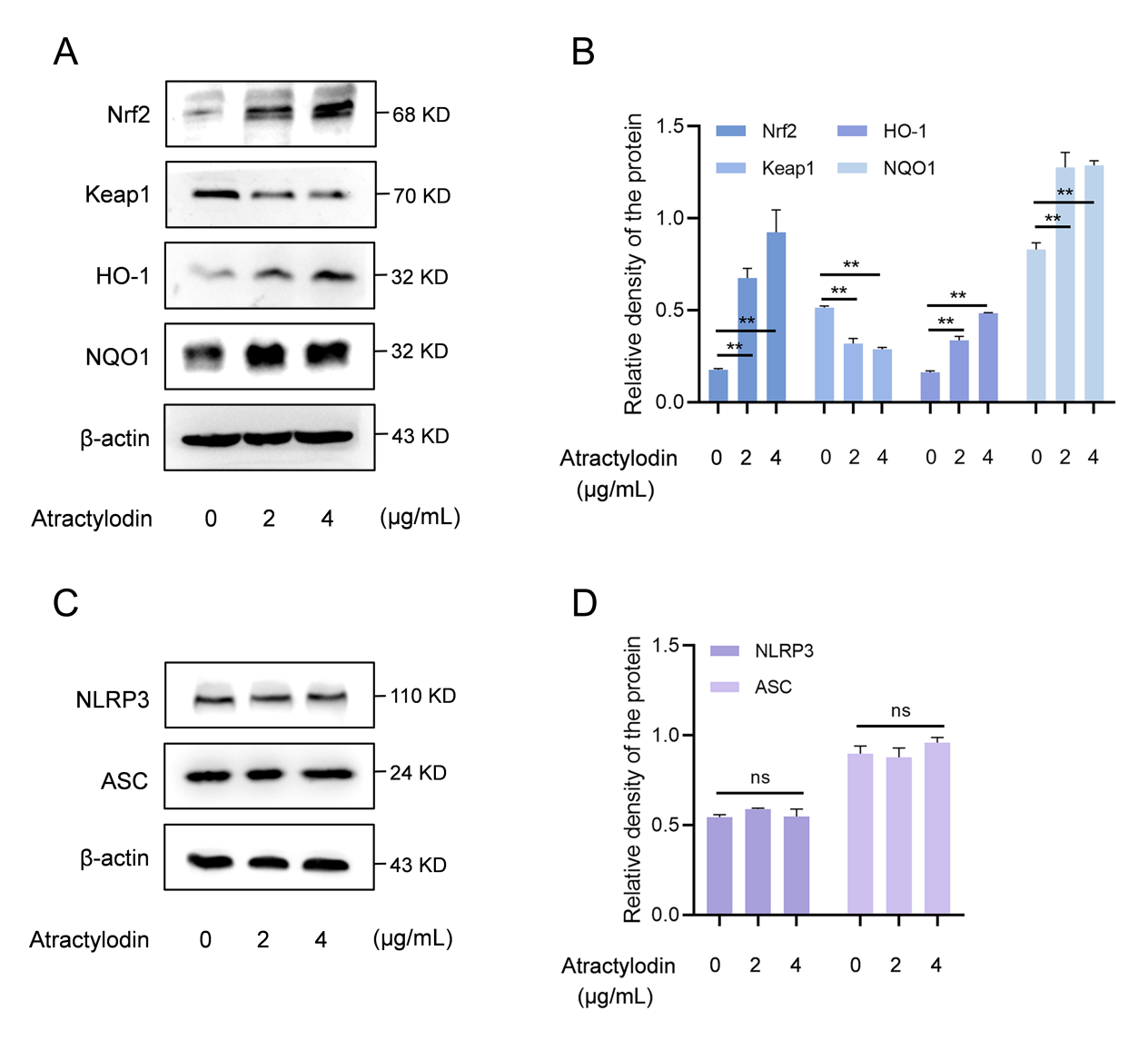


**Supplementary Figure 3 Effects of atractylodin on the Nrf2/HO-1 signaling pathway and NLRP3 inflammation in J774A.1 cells.** The effects of atractylodin (0, 2, 4 μg/mL) on the Nrf2/HO-1 signaling pathway **(A)** and NLRP3 signaling pathway **(C)** were examined by western blot analysis. **(B, D)** The relative density of protein was performed by densitometric analysis; β-actin was acted as an internal control. The data are representative of three biological replicates and expressed as ± SEM. ***P* < 0.01; ns, *P* > 0.05.
